# Postdischarge Care of Acute Kidney Injury Survivors: An Opportunity for Targeted Nurse and Pharmacist Interventions

**DOI:** 10.1053/j.akdh.2025.01.005

**Published:** 2025-03

**Authors:** Melanie M. Manis, Jessica L. Wallace, Emily F. Boyd, Kaleab Z. Abebe, Linda Fried, Paul M. Palevsky, Paul T. Conway, Edward J. Horwitz, Kathleen D. Liu, Chirag R. Parikh, Emilio Poggio, Edward D. Siew, Javier A. Neyra, Matthew R. Weir, F. Perry Wilson, Sandra L. Kane-Gill

**Affiliations:** Department of Medicine, Division of Nephrology, University of Alabama at Birmingham, Birmingham, AL; Department of Pharmacy and Pharmaceutical Sciences, Lipscomb University College of Pharmacy, Nashville, TN; Division of Nephrology and Hypertension, Vanderbilt University Medical Center, Nashville, TN; Division of Nephrology, Tennessee Valley Healthcare System (TVHS), Veterans Health Administration, Nashville, TN; Division of Nephrology and Hypertension, Vanderbilt University Medical Center, Nashville, TN; Center for Clinical Trials and Data Coordination, Division of General Internal Medicine, University of Pittsburgh School of Medicine; Renal-Electrolyte Division, University of Pittsburgh School of Medicine; Kidney Medicine Section, VA Pittsburgh Healthcare System; Renal-Electrolyte Division, University of Pittsburgh School of Medicine; Kidney Medicine Section, VA Pittsburgh Healthcare System; Policy and Global Affairs, American Association of Kidney Patients (AAKP), Tampa, FL Tampa, FL; Department of Nephrology, Metrohealth Medical Center, Cleveland, OH; Departments of Medicine and Anesthesia, University of California, San Francisco, CA; Department of Medicine, Division of Nephrology, Johns Hopkins University School of Medicine, Baltimore, MD; Department of Nephrology and Hypertension, Glickman Urological and Kidney Institute, Cleveland Clinic, Cleveland, OH; Division of Nephrology and Hypertension, Vanderbilt University Medical Center, Nashville, TN; Division of Nephrology, Tennessee Valley Healthcare System (TVHS), Veterans Health Administration, Nashville, TN; Department of Medicine, Division of Nephrology, University of Alabama at Birmingham, Birmingham, AL; Division of Nephrology, Department of Medicine, University of Maryland School of Medicine, Baltimore, MD; Clinical and Translational Research Accelerator, Department of Medicine, Yale School of Medicine, New Haven, CT; Department of Pharmacy and Therapeutics, School of Pharmacy, University of Pittsburgh, Pittsburgh, PA; Department of Pharmacy, UPMC, Pittsburgh, PA; Department of Critical Care Medicine, Program of Critical Care Nephrology, Pittsburgh, PA

**Keywords:** Acute kidney injury, Pharmacists, Nurses, Transitional care, Telemedicine

## Abstract

The incidence of acute kidney injury (AKI) is increasing, and with it, the population of individuals requiring post-AKI care. Post-discharge follow-up for AKI survivors is recommended within 90 days of an AKI episode to promote kidney recovery and potentially prevent progression of kidney disease. However, timely postdischarge care is often lacking or fragmented and poses a missed opportunity to prevent long-term complications of this condition. Suggested elements of follow-up care begin with a scheduled appointment with a physician and involve a bundled approach to care with health care providers’ communicating across sites, remote patient monitoring devices, review of medications, education, access, kidney care evaluation, and interdisciplinary collaboration to achieve these patient care goals. This article provides an overview of guidance documents for post-AKI care and the roles of the nurse and pharmacist as part of an interdisciplinary team in postdischarge care after a patient incurs an episode of AKI.

Acute kidney injury (AKI) occurs in 15–20% of hospitalized patients. Notably, the incidence of AKI is increasing and thus the need for post-AKI care.^1,2^ Patients with AKI are at risk of developing recurrent AKI, acute kidney disease (AKD), chronic kidney disease (CKD), and end-stage kidney disease requiring dialysis, cardiovascular disease, and debilitation with poor health-related quality of life following a prolonged hospitalization with need for acute rehabilitation.^3^ Each year approximately 1.6 million Americans progress from AKI to CKD.^4^ Even those patients with an apparent reversible AKI prior to hospital discharge have a significant risk of developing de novo CKD.^5^ The transition to CKD can include AKD, as AKD is a decline in kidney function lasting for less than or equal to 3 months.^6^ Six months after a drug-associated AKI event, kidney damage persists in 70% of cases.^7^ Exposure to one or more potentially nephrotoxic drugs presents an increased risk of developing de novo or progressive CKD for AKI survivors.^8^ Timely, postdischarge care after an episode of AKI could curtail worsening kidney health and unnecessary burdens on patients, caregivers, and the overall health care system.

It is unfortunate that post-AKI care is poor given the substantial negative consequences. In Medicare patients, only 13% saw a nephrologist within 90 days after an AKI episode.^3,9^ Additionally, many individuals with AKI were not aware they had the condition, making it less likely that they would seek follow-up.^10–14^ The lack of postdischarge AKI care is considered a missed opportunity to prevent chronic disease and complications.^15^ Evidence-based best practices for postdischarge care after an inpatient AKI event are unknown.

The reasons AKI survivors do not seek care, in addition to their lack of knowledge about kidney disease, include the feeling of hospital-related fatigue, a concern for adding more doctors to their care team, and the travel time to receive in-person care.^16^ Other individuals have challenges because post-AKI care co-ordination is lacking.^17^ These are opportunities for a nurse to provide supportive care, help with medical access, education, and coordinate AKI survivors’ follow-up care with the goal of improving patient outcomes.

Almost 5 million ambulatory care visits occur every year due to adverse drug events, and often these are associated with preventable medication errors.^18^ Complex medication regimens that require dose adjustments or drug discontinuation in the presence of reduced kidney function is a risk for medication errors in post-AKI survivors. A lack of an accountable clinician at hospital discharge to manage drug-related issues during transition of care is cited as a possible reason for missed medication errors.^19^ Patient-pharmacist interactions to enhance medication access, safety, and monitoring are well established. It is expected that AKI survivors are a vulnerable population that could benefit from pharmacist oversight during transitional care.^20,21^

This article provides an overview of the guidance documents for post-AKI care and the potential roles of the nurse and pharmacist as part of an interdisciplinary team in postdischarge care after a patient experiences an episode of AKI.

## OPINION-BASED GUIDANCE DOCUMENTS FOR POST-AKI/AKD CARE AND OPPORTUNITIES FOR NURSE AND PHARMACIST INTERVENTIONS

Despite the importance of post-AKI care, recommendations and supporting evidence are sparse. The 2012 KDIGO guidelines suggest an evaluation “within 3 months to assess for resolution, new-onset, or worsening of pre-existing CKD.”^22^ Since 2012, several groups have recognized the lack of data on supportive patient care and have aimed to improve postdischarge AKI care processes by proposing bundled frameworks and quality indicators to drive future research. The bundled frameworks are summarized in Table 1 and highlight the potential role for nurse and pharmacist interventions in the care process.^23–27^

**Figure 1.**
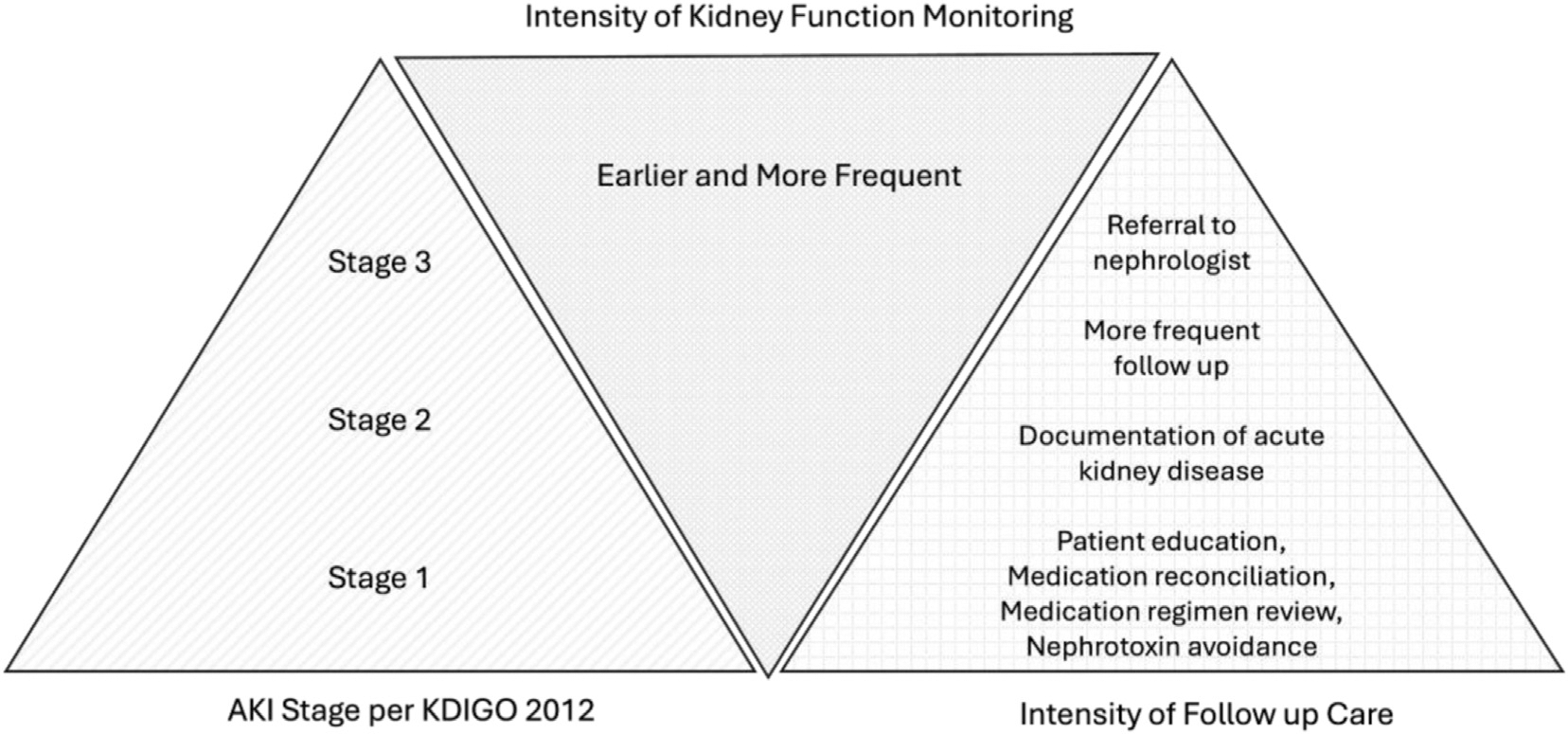
A layered approach to follow-up care for patients with acute kidney disease. Modified from Acute Dialysis Quality Initiative 16; www.adqi.org. Abbreviations: AKI, acute kidney injury; KDIGO, kidney disease: improving global outcomes.

In 2017, the Acute Disease Quality Initiative (ADQI) consensus statement providing practice guidance for AKD management and renal recovery was released.^23^ In response to the lack of data for timing, frequency, and methods of evaluating kidney function following an episode of AKI, the group proposed a “layered” approach for follow-up with intensity of care based on the severity of kidney dysfunction and the risk of morbidity and mortality (Fig 1). Specific suggestions for drug management in patients with AKD were also provided supporting pharmacists’ involvement in the care of patients with AKD.

In 2019, ADQI published Quality Improvement Goals for AKI. The authors proposed key elements of an appropriate post-AKI/AKD Care Bundle that included components of KAMPS: kidney function assessment-advocacy-medications-pressure-sick day protocols for patients with AKI and WATCH-ME: weight assessment-access-teaching-clearance medication-hypotension for patients with AKI who require dialysis.^24^ The authors acknowledged the need for future research focused on optimal management strategies for each facet of the KAMPS/WATCH-ME bundles and the need for validation of the bundles. Notably, this is the only post-AKI/AKD care bundle that includes sick day protocols. A scoping review of 74 sick day medication guidance documents indicates consensus on how to apply this guidance is lacking, so inclusion in post-AKI care is questionable.^25^ Still, many aspects of KAMPS/WATCH-ME would benefit from nurses’ and pharmacists’ participation to facilitate implementation.

The Optimum Care of AKI Survivors Not Requiring Dialysis after Discharge Report released in January 2024 by the American Society of Nephrology AKINow Recovery workgroup discussed the challenges and opportunities for post-AKI care.^26^ This report emphasized 6 domains: transitional care, education, collaborative care delivery, evidence and guidance for diagnostic and therapeutic interventions, and digital health applications. Ultimately, similar suggestions were made by AKINOW and previous guidance documents indicating that more research is needed to drive patient-centered, evidence-based care for AKI survivors. Interestingly, 5 and 8 of the 12 AKINow recommendations are prospects for nurses’ and pharmacists’ contributions, respectively. The AKINow Recovery workgroup is currently working on tools to assist in care continuity at the time of hospital discharge.

## POTENTIAL ROLES FOR A NURSE NAVIGATOR IN POSTDISCHARGE AKI CARE

### Postdischarge Care by Nurses

The nurse’s role in a transitional care capacity has been termed “nurse case manager,” “transition coach,” “nurse liaison,” and “nurse navigator.” This role of the nurse first originated in oncology to help bridge the care gap with the nurse serving as the main point of contact through the patient’s care trajectory, leading the coordination of care, and providing education to help patients throughout their health care journey.^28,29^ Nurses have demonstrated to be a beneficial member of an interdisciplinary team providing postdischarge care co-ordination in various other conditions. In a general population of hospitalized patients, a randomized trial demonstrated the value of a nurse “transition coach” to provide postdischarge care support including home visits and telephone calls to reduce hospital readmissions at 30 days (8.3 vs 11.9, P =.048) and 90 days (16.7 vs 22.5, P =.04). The 4 components of the intervention included (1) patient’s self-management of medications; (2) timely postdischarge follow-up; (3) self-monitoring of “red flags” and at-home response, and (4) patient-maintained medical record to aid in transitions of care.^30^ This intervention is easily translated to the AKI survivor population. Further, postdischarge transitional care nursing models are well described in heart failure patients, who are at high risk for readmission and could also be applied to AKI survivors.^31^

### Postdischarge AKI Care by Nurses

AKI survivors face similar risk of rehospitalization with complexity of multimorbidity, often including concomitant heart failure, and prolonged hospitalizations, which warrant a nurse dedicated to coordinating care following discharge to arrange for critical follow-up visits given the coexistence of often both cardiac and renal complications of hospitalization and need for close monitoring of this dynamic patient. Beyond care coordination, education is also an essential role of the nurse. With 80% of patients estimated to be unaware of AKI diagnosis at time of hospital discharge, education by the nurse and continual reinforcement of that education is invaluable to promote kidney health.^11^ The role of the nurse as part of the interdisciplinary care team for AKI survivors supports nurses as the key educators at discharge utilizing a variety of educational resources (eg, video on AKI, verbal discussion, written materials) with standardized teach-back questions to verify understanding.^32^ Important components of AKI education identified by the nurses in this study included the potential for recovery and risk of reinjury. Furthermore, nurses may be used in the clinic setting postdischarge. In an interdisciplinary educational study of postdischarge AKI clinic patients, nurses engaged in a 10–15-minute educational interaction at the first clinic visit to identify what was unknown by the patient about AKI using open-ended questions and self-reflection of the hospital experience.^33^ The nurse answered questions and concerns and provided an AKI educational handout available from the National Kidney Foundation. The patient-nurse interaction was followed by patient interactions with the pharmacist and a physician. This was the first study to evaluate an educational effort by the interdisciplinary team and reported a significant improvement in patients’ self-perceived knowledge.

The next vital role of the nurse is close objective monitoring of blood pressure and body weight for volume management, which is an important component of care for AKI survivors. Often antihypertensives and/or diuretics are held or dose adjusted during hospitalization, making close monitoring necessary to avoid complications. Remote monitoring of vital signs by nurses in postdischarge AKI care has been described in the literature and may offer improved access to monitoring care.^34^ In a general patient population with various health conditions including most commonly COVID-19, heart failure, or hypertension, a nurse-led remote patient monitoring program allowed for improved connection with patients and access to care, resulting in a lower 30-day, all-cause hospitalization rate (13.7% vs 18.0%, *P* = 0.01), and high patient satisfaction.^35^ Beyond hospitalization rates and satisfaction, outcomes such as improved blood pressure control have been observed in the literature with remote monitoring programs implemented amidst the COVID-19 pandemic involving a multidisciplinary care team including nurse practitioners.^36^ AKI survivors could highly benefit from the application of this nurse-led remote monitoring care model given the need for ongoing assessment of blood pressure and weights.

When the nurse’s role on the interdisciplinary care team for post-AKI care is described in the literature, the role of the nurse is focused on a single role such as education or care co-ordination.^33,34,37^ Literature describing a comprehensive, multifaceted role of the nurse in postdischarge AKI care is limited. In a nonrandomized study of stage 2–3 AKI survivors after hospital discharge, Singh and colleagues outlined an AKI rehabilitation program that featured a nurse performing multiple different roles, which was associated with a reduced risk of mortality or rehospitalization at 30 days (odds ratio, 0.41; 95% confidence interval, 0.16–0.93) and 90 days (odds ratio, 0.52; 95% confidence interval, 0.25–1.05).^38^ In conclusion, nurses are crucial members of the care team to aid in providing postdischarge AKI care, with opportunities to enhance outcomes through roles such as care coordination, patient education, and monitoring. However, further pragmatic studies are needed to prospectively assess the impact of nurses’ contributions in AKI survivors.

## POTENTIAL ROLES FOR A PHARMACIST IN POSTDISCHARGE AKI CARE

### Postdischarge Care by Pharmacists

Pharmacists are widely engaged in transitional care of patients when resources are available to do so. A central activity is conducting medication reconciliation to identify discrepancies between hospital discharge prescriptions and a patient’s prehospital medications, thus preventing medication errors and adverse drug events. Transitional care pharmacists also educate patients and caregivers about proper medication use, side effects, lifestyle changes, and medication adherence and empower patients to manage their health independently. Studies have demonstrated that pharmacist-led interventions can reduce medication errors by at least 37%, decrease 30-day readmission rates by up to 20%, and improve disease management in conditions like heart failure, asthma, and diabetes.^39–42^ In patients with kidney injury, pharmacists’ involvement in transitional care is especially relevant given the complex medication challenges in this patient population.

### Postdischarge AKI Care by Pharmacists

Pharmacists are the medication experts of the interdisciplinary care team with advanced pharmacokinetic knowledge including drug elimination and its impact on dosing in kidney dysfunction, as well as mechanisms of nephrotoxicity and management. Beyond kidney health considerations, pharmacists are trained to provide comprehensive medication management (CMM), patient education, and communication hand-off to other health care providers.^43^

Several studies have demonstrated the value of a pharmacist as part of the interdisciplinary team when providing postdischarge care.^44,45^ In an end-stage kidney disease Seamless Care Organization, pharmacists participated in an interdisciplinary medication therapy management intervention that identified 5466 medication therapy problems and reduced hospital readmissions (*P* < 0.001). In this intervention, the nurse provided an initial medication reconciliation while the pharmacist conducted a secondary medication review and created an action plan that was communicated to the nephrologist.^45^ Pharmacists have had a positive impact on clinical outcomes including readmission rates and death at 90 days and medication safety in other disease states, leading to the recognition of clinical pharmacists as an integral part of the patient care team by several organizations including the American Heart Association/American College of Cardiology/Heart Failure Society of America, the American Diabetes Association, the Society of Critical Care Medicine, and the Organ Procurement and Transplantation Network.^33,46–49^

AKI survivors are recognized as an ideal patient population that would benefit from pharmacists’ hypervigilance due to nuances in complex medication management as stated above.^50^ Nephrologists and primary care physicians requested clinical pharmacists’ engagement in medication management when caring for AKI survivors to overcome barriers in medication mismanagement and to provide medication-related guideline adherence.^51^ The ADQI Quality Improvement Goals for AKI recommend that pharmacists be included as a member of the dedicated interdisciplinary team and that pharmacists perform medication reconciliations, reviews, and management as part of the post-AKI/AKD kidney health bundle.^24^ Additional support for an elevated role of pharmacists on kidney interdisciplinary care teams and within national kidney care models, along with CMM for people with kidney disease, has gained support of kidney professionals and patients through the broad-based Optimizing Kidney Health through Optimal Medical Management collaborative and growing interest from the Centers for Medicare and Medicaid Services.^52^

**Figure 2.**
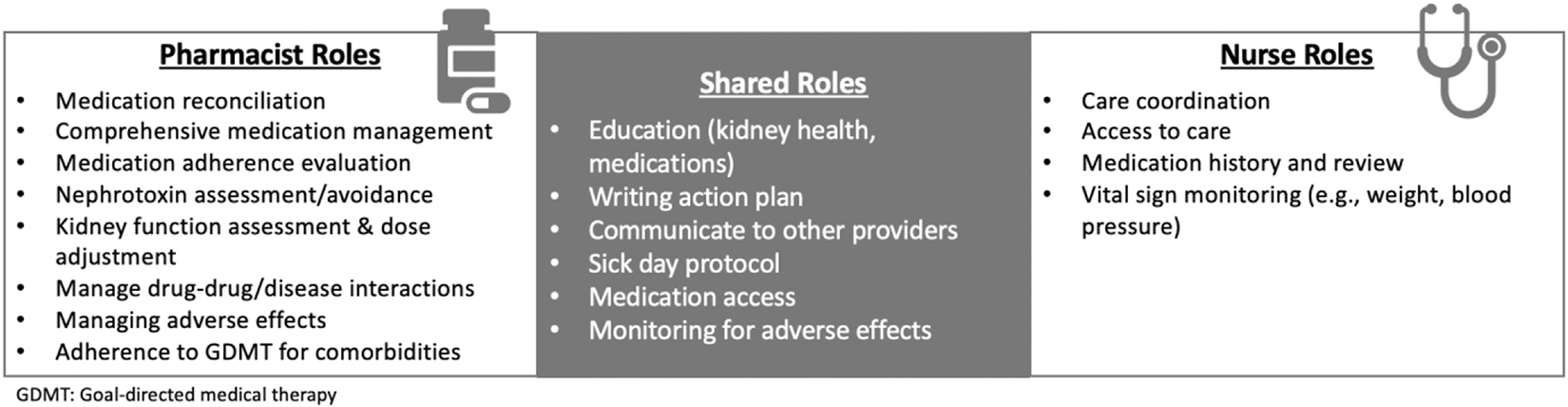
Proposed shared and individual roles for the pharmacist and nurse in postdischarge AKI care. Abbreviations: AKI, acute kidney injury; GDMT, goal-directed medical therapy.

Unfortunately, the specific role of the pharmacist in providing postdischarge AKI care is difficult to elucidate, as they are often embedded in care teams where members’ activities target a variety of outcomes and key details are often missing or not fully described. But pharmacist-centric studies are being investigated, so we may have a clearer assessment in the future.^53^ A proposal for the role of a pharmacist caring for patients with AKD was published in 2017 and included enhancing health literacy about medication management, managing drug-drug interactions, avoiding or cautiously restarting nephrotoxins post-AKI, encouraging monitoring of kidney function, and managing diabetes and hypertension as part of the care team. Proposed activities occurred during hospitalization, at the time of discharge, and postdischarge and specifically encouraged telemedicine interactions and/or meeting patients in the post-AKI ambulatory clinics.^48^ A recently published scoping review evaluated the role of the pharmacist in providing postdischarge care to patients with AKI and CKD.^54^ Four postdischarge AKI care team models with pharmacists were identified.^33,37,53–56^ Although the role of the pharmacist varied significantly among interdisciplinary care team models, a medication regimen review was consistently performed by a pharmacist. Medication reconciliation and writing action plans were also commonly performed by the pharmacist. Still, it is proposed the pharmacist’s roles should expand beyond medication review and reconciliation to include CMM. Kidney function assessment with drug dosing modifications, education on medications and kidney disease, as well as assistance with medication access and adherence, and finally communication with other health care team members were also proposed.^48^ Figure 2 depicts proposed roles specific to the pharmacist based on their medication expertise.

## CHALLENGES FOR NURSES AND PHARMACISTS IN THE ROLE OF AKI SURVIVOR CARE

Widespread use of nurses and pharmacists as part of an interdisciplinary team for AKI survivors’ care requires recognizing these patients as a priority so resources can be dedicated accordingly. In general, postdischarge patient care is important, so giving precedence to AKI survivors requires explicit evidence to support this narrow focus. A comprehensive team dedicated to postdischarge care of AKI survivors seems logical but requires a full understanding of the impact on patient outcomes to garner support. There has been at least one study suggesting a lack of benefit associated with pharmacist involvement in the care of CKD patients after hospitalization, but the intervention was limited to medication reconciliation and not a comprehensive medication review and did not focus on AKI survivors.^57^ Other studies have demonstrated the feasibility and potential for positive outcomes including better blood pressure control, improved urine albumin to creatinine ratio, improved kidney health literacy, and a reduction in 30-day rehospitalization and mortality having a nurse and pharmacist as part of the interdisciplinary team for AKI survivors.^37,38,54,58,59^ Still, other challenges include the shortage of nurses and pharmacists in the workforce to complete these duties and the financial support to secure dedicated positions.

## ONGOING RESEARCH: CARING FOR OutPatiEnts AFTER ACUTE KIDNEY INJURY (COPE-AKI)

COPE-AKI is a randomized, prospective trial funded through National Institutes of Health/National Institute of Diabetes & Digestive & Kidney Diseases and seeks to provide evidence for many of the suggestions made in the guidance documents. Directed by AKI patient survivors’ insights, COPE-AKI study collaborators established a care bundle that includes communication/coordination, remote objective monitoring, review of medications, education, access, kidney care evaluation, and interdisciplinary collaboration of a study physician, nurse navigator, and pharmacist (Table 2).^27,60–62^ The nurse serves an integral role as the primary contact with the patients to co-ordinate postdischarge care. The nurse educates on self-monitoring and use of remote monitoring equipment (blood pressure cuff and weight scale). Postdischarge, the nurse navigator monitors the patient’s condition, facilitates scheduling of routine and ad-hoc medical follow-up, facilitates adherence with prescribed medical care and follow-up appointments, provides education, and serves as a resource for AKI-related management, along with ensuring psychosocial support.^27^ The pharmacist also plays an important role by performing a postdischarge telemedicine visit with the patient and completing a medication reconciliation plus a comprehensive medication regimen review. The pharmacist evaluates appropriate drug dosing based on kidney function, nephrotoxic burden, and drug-drug or drug-disease interactions. Education related to kidney injury, medications to avoid, and medication adherence is also provided. The pharmacist also assists with medication access and barriers to care.

Results from this study may help further define the role of the nurse and pharmacist in post-AKI care. This is the first randomized prospective study to include a multifaceted nurse role as part of the interdisciplinary care team for postdischarge AKI care. Pragmatic studies evaluating kidney disease patients are increasing with inclusion of both pharmacists and nurse on the multidisciplinary care team.

## SUMMARY

Opportunities to improve care of AKI survivors after hospital discharge include supporting appropriate provider follow-up to ensure CMM and monitoring of kidney function. Postdischarge AKI care by an interdisciplinary team has been proposed to improve outcomes in these individuals. Nurses could play a critical role in care coordination for post-AKI patients to improve medical access along with providing ongoing patient communication to deliver education about kidney health and monitoring of the patient’s condition. AKI survivors often have multimorbidity and complex medication regimens at hospital discharge; pharmacists are essential in performing medication reconciliation, medication regimen reviews, and education. By coordinating care across different settings, interdisciplinary teams ensure smooth transitions of patients and prevent gaps in care that could lead to readmissions. While many guidance documents propose the optimal care of patients with AKI and AKD, further research is needed to evaluate the impact of these care bundles, assess the impact on patient outcomes, determine effective implementation strategies, and inform future policy proposals.

## Figures and Tables

**Table 1.** Recommendations in Guidance Documents for Post-AKI/AKD Care

	2019 ADQI Post-AKI/AKD Bundle^24^		2024 AKINow Recovery Workgroup^26^	Caring for OutPatiEnts after AKI (COPE-AKI)^27^
	KAMPS for Patients With AKI	WATCH-ME for Patients With AKI Requiring Dialysis		

Documentation of AKI/AKD			✔	✔
Kidney function assessment	✔	✔	✔	✔
Education of patient/caregiver*†	✔	✔	✔	✔
Sick day protocol*†	✔			
Medication reconciliation†	✔	✔		✔
Medication management†	✔	✔	✔	✔
Nephrotoxin avoidance†	✔	✔		✔
Adjust medications based on kidney function†	✔	✔	✔	✔
RASi/MRA/diuretic assessment†	✔	✔	✔	✔
Blood pressure targets*	✔	✔	✔	✔
Weight assessment†	✔	✔		✔
Communicate with other providers*†	✔	✔	✔	✔
Digital health applications			✔	
Telehealth			✔	✔
Nephrology referral				✔
Access (arteriovenous)		✔		
Multidisciplinary collaboration*†			✔	✔
Patient-centered care plans*†			✔	✔

Abbreviations: KAMPS, kidney function assessment-advocacy-medications-pressure-sick day protocols; WATCH-ME, weight assessment-access-teaching-clearance medication-hypotension; AKI, acute kidney injury; AKD, acute kidney disease; ADQI, Acute Disease Quality Initiative; COPE-AKI, Caring for OutPatiEnts after Acute Kidney Injury.

* Opportunities for nurse interventions.

† Part of the nephrologist discharge assessment.

**Table 2.** COPE-AKI Care Bundle for Patients Experiencing Stage II and Stage III AKI During Hospitalization^17^

**C**ontinuous communication with the patient following hospital discharge, communication between acute care providers and postdischarge care providers (eg, primary care providers, nephrologist) and **c**oordination of patient care by nurse navigator
**O**bjective monitoring (ie, weight, blood pressure) by nurse navigator and monitoring of kidney function during postdischarge care provider visits
**P**harmacist review of medications (ie, medication regimen review*, medication reconciliation†, and medication management‡)
**E**ducation about AKI episode, kidney health, and avoidance of nephrotoxins and **E**nhanced psychosocial support
**A**ccess to postdischarge health care needs including an appointment with primary care physician and/or nephrologist, access to medications, etc
**K**idney care evaluation by nephrologist to conduct a discharge assessment to triage and make recommendations for follow-up care
**I**nterprofessional collaboration of care optimized

Abbreviation: AKI, acute kidney injury.

* Medication therapy review: a systematic process of collection of patient-specific information, assessing medication therapies for the identification of medication-related problems, development of a prioritized list of medication-related problems, and creation of a plan to resolve them”.^60^

† Medication reconciliation: process of identifying most accurate patient medication list (including name, dosage, frequency, and route) from a comparison of the medical record to an external list of medications obtained from a patient, hospital, and/or other provider.^61^

‡ Medication therapy management: distinct service or group of services that optimize therapeutic outcomes for individual patients.^62^

## CLINICAL SUMMARY

- Several guidance documents propose optimal care strategies for survivors of hospitalized acute kidney injury, but the impact of bundled care provided by an interdisciplinary team requires an evaluation of impact on patient outcomes.
- Nurses champion the role of education and care coordination to improve knowledge of kidney health and medical access along with ongoing patient communication to monitor the patient’s condition.
- Pharmacists, as the medication experts, are essential in performing medication reconciliation and medication regimen reviews.
